# Interferon-β attenuates lung inflammation following experimental subarachnoid hemorrhage

**DOI:** 10.1186/cc9232

**Published:** 2010-08-23

**Authors:** Pieter M Cobelens, Ivo ACW Tiebosch, Rick M Dijkhuizen, Peter H van der Meide, René Zwartbol, Cobi J Heijnen, Jozef Kesecioglu, Walter M van den Bergh

**Affiliations:** 1 Department of Intensive Care Medicine, University Medical Center Utrecht, Heidelberglaan 100, Utrecht, 3584 CX, The Netherlands; 2 Laboratory of Neuroimmunology and Developmental Origins of Disease (NIDOD), University Medical Center Utrecht, Lundlaan 6, Utrecht, 3584 EA, The Netherlands; 3 Biomedical MR Imaging and Spectroscopy Group, Image Sciences Institute, University Medical Center Utrecht, Yalelaan 2, Utrecht, 3584 CM, The Netherlands; 4 Cytokine Biology Unit, Central Laboratory Animal Institute, Utrecht University, Bolognalaan 50, Utrecht, 3508 TD, The Netherlands; 5 Department of Intensive Care Medicine, Academic Medical Center, University of Amsterdam, Meibergdreef 9, Amsterdam, 1105 AZ, The Netherlands

## Abstract

**Introduction:**

Aneurysmal subarachnoid hemorrhage (SAH) affects relatively young people and carries a poor prognosis with a case fatality rate of 35%. One of the major systemic complications associated with SAH is acute lung injury (ALI) which occurs in up to one-third of the patients and is associated with poor outcome. ALI in SAH may be predisposed by neurogenic pulmonary edema (NPE) and inflammatory mediators. The objective of this study was to assess the immunomodulatory effects of interferon-β (IFN-β) on inflammatory mediators in the lung after experimental SAH.

**Methods:**

Male Wistar rats were subjected to the induction of SAH by means of the endovascular filament method. Sham-animals underwent sham-surgery. Rats received IFN-β for four consecutive days starting at two hours after SAH induction. After seven days, lungs were analyzed for the expression of inflammatory markers.

**Results:**

SAH induced the influx of neutrophils into the lung, and enhanced expression of the pulmonary adhesion molecules E-selectin, inter-cellular adhesion molecule (ICAM)-1, and vascular cell adhesion molecule (VCAM)-1 compared to sham-animals. In addition, SAH increased the expression of the chemokines macrophage inflammatory protein (MIP)-1α, MIP-2, and cytokine-induced neutrophil chemoattractant (CINC)-1 in the lung. Finally, tumor necrosis factor-α (TNF-α) was significantly increased in lungs from SAH-animals compared to sham-animals. IFN-β effectively abolished the SAH-induced expression of all pro-inflammatory mediators in the lung.

**Conclusions:**

IFN-β strongly reduces lung inflammation after experimental SAH and may therefore be an effective drug to prevent SAH-mediated lung injury.

## Introduction

Aneurysmal subarachnoid hemorrhage (SAH) affects patients at a relatively young age and has an incidence of 5 to 7 per 100,000 person-years. SAH carries a bad prognosis with a case fatality rate of 35% [1]. Systemic complications including cardiac failure, pneumonia, and pulmonary edema, occur in more than half the patients with SAH, and are an important contributor to poor outcome [2].

Acute lung injury (ALI) is commonly associated with brain injury and has an incidence between 5 and 30% in SAH patients [2-5]. The development of ALI after SAH is an independent predictor of increased mortality and poor neurological outcome in patients suffering from SAH. One of the major components of ALI is the formation of pulmonary edema. Neurogenic pulmonary edema (NPE) is a life-threatening complication in patients suffering from SAH and is associated with a three-fold increase in poor outcome [6].

The proposed etiology of NPE is a massive sympathetic discharge following severe brain injury leading to intra-alveolar accumulation of protein-rich edema fluid and hemorrhage. The pathogenesis of NPE is still incompletely understood. A well-established theory on the pathophysiology of NPE is the *blast-theory*, which proposes combined hydrostatic and high permeability mechanisms for the formation of NPE due to the sympathetic storm caused by an acute increase in intracranial pressure [7]. Recently, it has been suggested that a systemic inflammatory response may also play a pivotal role in the development of pulmonary dysfunction after traumatic brain injury [8,9]. The effects, however, of SAH on inflammatory mediators in secondary organs including the lung are still unknown.

Current therapy is mainly supportive since no effective drugs are available to treat NPE. Mechanical ventilation (MV) is necessary in the majority of patients to assure adequate oxygenation. However, MV can also contribute to lung injury, called ventilation-induced lung injury (VILI) [10]. A staggering 40% of SAH patients on MV develop VILI [11].

Interferon-β (IFN-β) is a small protein with immunomodulatory properties that has been approved for treatment of multiple sclerosis. It has been shown that IFN-β decreases pro-inflammatory cytokines and inhibits the migration of lymphocytes across the blood-brain barrier by decreasing the expression of chemokines and adhesion molecules on endothelial cells [12].

The aim of the present study was to investigate whether experimental SAH contributes to lung inflammation. Moreover, we examined the effects of IFN-β treatment on the expression of inflammatory mediators in the lung associated with brain injury in a rat model of SAH.

## Materials and methods

### Animals

The experiments were performed in accordance with international guidelines and approved by the experimental animal committee of the Academic Biomedical Center Utrecht. Male Wistar rats (weighing 320 to 350 g) were obtained from Harlan CPB (Horst, The Netherlands) and randomly assigned to the different treatment groups. Both the executers of the experiments and of the statistical analysis were blind for randomization.

### Experimental SAH-model

Rats were intubated under gaseous anesthesia (65% air/33% oxygen/2% isoflurane) and mechanically ventilated for a maximal 90 minutes in a pressure controlled time-cycled mode, at a fractional inspired oxygen concentration (FiO_2_) of 0.5, inspiration to expiration (I/E) ratio of 1:1 and peak inspiratory pressure of 10 cmH_2_O. To maintain normocapnia, the respiratory rate was set at 55 breaths per minute. The left external carotid artery (ECA) was ligated and cut, while the ipsilateral internal carotid artery (ICA) and communal carotid artery were temporarily clipped. A sharpened 4.0 prolene suture was introduced through an opening in the ligated left ECA and distally advanced through the ICA until the suture perforated the intracranial bifurcation of the ICA. In sham-animals, the suture was withdrawn prior to perforating the ICA. The presence of subarachnoidal blood was confirmed with magnetic resonance imaging.

Animals were treated for four consecutive days with subcutaneous injections of 1.75 × 10^6 ^U/kg IFN-β (U-Cytech, Utrecht, The Netherlands) (SAH-animals: *N *= 6; sham-animals: *N *= 4) or saline (SAH-animals: *N *= 7; sham-animals: *N *= 4) starting at two hours after SAH. Treatment dosage and time of treatment were based on previous results of Veldhuis *et al*. where IFN-β proved to be clinically efficacious by reducing the influx of inflammatory cells into the brain and reducing infarct volume in a rat model of ischemic stroke when started up to six hours after the induction of stroke [13]. Since we wanted to initiate treatment as soon as possible, but with respect to a realistic clinical moment at which patients are admitted to a hospital, we decided to start treatment at two hours after the induction of stroke.

### Preparation of tissue homogenates

Lungs were removed at seven days post-SAH. Tissues were pulverized using a liquid nitrogen-cooled mortar and pestle and stored at -80°C for further analysis.

### Myeloperoxidase (MPO) assay

MPO activity was determined as described previously [14]. Briefly, pulverized tissues were homogenized in 50 mM HEPES buffer (pH 8.0), centrifuged and pellets were rehomogenized in H_2_O/0.5% cetyltrimethylammonium chloride (CTAC; Merck, Darmstadt, Germany). After centrifugation, supernatants were diluted in 10 mM citrate buffer (pH 5.0)/0.22% CTAC. Substrate solution containing 3 mM 3',5,5'-tetramethylbenzidine dihydrocloride (TMB; Sigma-Aldrich, Steinheim, Germany), 120 μM resorcinol (Merck) and 2.2 mM H_2_O_2 _in distilled water was added. Reaction mixtures were incubated for 20 minutes at room temperature and stopped by 4 M H_2_SO_4_, followed by determination of optical density at 450 nm. MPO activity of a known amount of MPO units (Sigma-Aldrich) was used as reference.

### Quantitative real-time reverse transcriptase (RT)-PCR analysis

Total RNA was isolated from pulverized tissues with TRIzol^® ^reagent (Invitrogen, Paisley, UK). cDNA was synthesized from total RNA with SuperScript Reverse Transcriptase kit (Invitrogen). Quantitative real-time RT-PCR reaction was performed with iQ5 Real-Time PCR Detection System (Biorad, Hercules, CA, USA) using rat primers for TNF-α, macrophage inflammatory protein (MIP)-1α, MIP-2, cytokine-induced neutrophil chemoattractant (CINC)-1, E-selectin, inter-cellular adhesion molecule (ICAM)-1, and vascular cell adhesion molecule (VCAM)-1. Data were normalized for expression of internal controls, β-actin and GAPDH.

### Statistical analysis

Data are expressed as mean ± standard error of mean (SEM). All parameters were evaluated by one-way analysis of variance (ANOVA) with Least Significance Difference (LSD) post-test. *P*-values < 0.05 were considered statistically significant.

## Results

### Stability of the model

SAH was induced at Day 0 in male Wistar rats and treated for four consecutive days with IFN-β. At Day 7, placebo-treated rats showed a case fatality rate of 50%, whereas IFN-β-treated animals showed a case fatality rate of 63%, although this difference was not statistically significant. All animals that did not survive the protocol died within the first three days after the induction of SAH.

### IFN-β inhibits the influx of neutrophils into the lung

**Figure 1:**
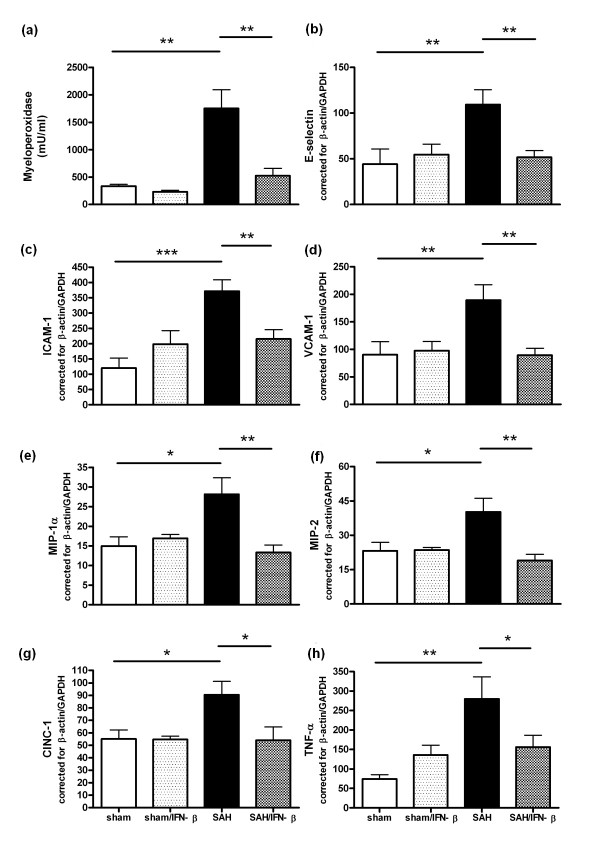
**SAH was induced in male Wistar rats**. Rats were treated with IFN-β for four consecutive days starting at two hours after the induction of SAH. Seven days post-SAH total lung homogenates were prepared and analyzed for inflammatory markers. **(a) **MPO activity corrected for the amount of protein. **(b-d) **Expression of endothelial activation markers E-selectin, ICAM-1, and VCAM-1. **(e-g) **Expression of chemokines MIP-1α, MIP-2, and CINC-1. **(h) **Expression of TNF-α. (b-h) Data are normalized for the expression of β-actin and GAPDH. Sham: *N *= 4; Sham/IFN-β: *N *= 4; SAH: *N *= 7; SAH/IFN-β: *N *= 6. *** *P *< 0.001, ** *P *< 0.01, * *P *< 0.05; SAH *vs *sham or SAH/IFN-β *vs *SAH. MPO, myeloperoxidase; MIP, macrophage-inflammatory protein; CINC, cytokine-induced neutrophil chemoattractant.

As a parameter for the number of infiltrating neutrophils we determined myeloperoxidase (MPO)-activity in total lung homogenates at Day 7 post-SAH. SAH-animals had a significant increase in MPO-activity compared to sham-animals (*P *< 0.01), which could be efficiently blocked by IFN-β (*P *< 0.01; Figure 1a).

### IFN-β inhibits the expression of endothelial activation markers and pulmonary chemokines

Because neutrophil influx is dependent on the activation of endothelial cells, we studied the effect of SAH on endothelial adhesion molecules in lung tissue. Compared to sham-animals, SAH increased pulmonary expression of E-selectin (*P *< 0.01), inter-cellular adhesion molecule (ICAM)-1 (*P *< 0.001), and vascular cell adhesion molecule (VCAM)-1 (*P *< 0.01). IFN-β normalized the expression of E-selectin (*P *< 0.01), ICAM-1 (*P *< 0.01) and VCAM-1 (*P *< 0.01) compared to saline-treated SAH-rats (Figure 1b-d).

To establish whether the attraction of neutrophils is correlated with the expression of chemokines, we next questioned whether SAH induced the expression of pulmonary macrophage inflammatory protein (MIP)-1α, MIP-2, and cytokine-induced neutrophil chemoattractant (CINC)-1. All chemokines were significantly increased in lungs from SAH-rats (*P *< 0.05). Treatment with IFN-β completely abolished the SAH-induced upregulation of the chemokines MIP-1α (*P *< 0.01), MIP-2 (*P *< 0.01), and CINC-1 (*P *< 0.05) (Figure 1e-g).

### IFN-β reduces pulmonary TNF-α expression

To determine whether SAH induced pro-inflammatory cytokine expression in the lung, we measured TNF-α expression. SAH resulted in a significant three-fold increase in pulmonary TNF-α expression compared to sham-animals (*P *< 0.01). IFN-β treatment significantly decreased the SAH-induced TNF-α expression to the levels observed in IFN-β treated sham-animals (*P *< 0.05; Figure 1h).

## Discussion

We report here that SAH induces the influx of neutrophils into the lung and expression of pulmonary adhesion molecules, chemokines, and TNF-α. More importantly, we are the first to show that IFN-β effectively abolishes the SAH-induced expression of all pro-inflammatory mediators in the lung.

SAH rats showed a case fatality rate of 50% after seven days. The mortality observed by us is common in experimental SAH-models and resembles other findings [15-17]. Treatment with IFN-β resulted in a case fatality rate of 63%, although this increase did not statistically differ from the placebo group.

Neutrophils play an essential role in the development of lung inflammation. We observed a four-fold increase in MPO, indicating that neutrophils are recruited to the lung following SAH. The influx of neutrophils involves a complex cascade of events. The early response cytokine, TNF-β, most probably initiates the inflammatory response by activating the endothelial cells resulting in increased chemokine expression and upregulation of adhesion molecules [18]. In support of this concept we showed increased expression of pulmonary TNF-α following SAH. In addition, SAH induced enhanced pulmonary expression of the endothelial adhesion molecules E-selectin, ICAM-1, and VCAM-1, which play a pivotal role in the rolling across the endothelium and firm adhesion of neutrophils to the endothelium, respectively [18]. Furthermore, we observed marked SAH-induced upregulation of the chemokines MIP-1α, MIP-2, and CINC-1, which are responsible for the chemotactic activity of neutrophils. Therefore, we conclude that SAH induces a pro-inflammatory environment in the lung which may represent an important risk factor for the development of NPE.

Although several groups reported the occurrence of lung injury after brain injury, the underlying mechanisms are largely unknown. It has been suggested that increased intracranial production of pro-inflammatory cytokines results in the release of systemic pro-inflammatory mediators, thereby promoting secondary organ injury [8]. Although this is a tempting hypothesis, we show here *de novo *synthesis of pro-inflammatory mediators, suggesting that spill-over of cytokines is not the primary cause of lung inflammation following SAH. A second proposed mechanism underlying secondary organ injury is increased capillary permeability elicited by catecholamines due to sympathetic nerve stimulation after brain injury [7]. Catecholamines can activate the transcription factor NFκB in macrophages thereby promoting the production of TNF-α chemokines, and adhesion molecules [19,20]. Therefore, we propose that sympathetic activation of the lung could have led to the local release of cytokines and chemokines in our model.

The major finding of our study was that IFN-β treatment strongly attenuates the SAH mediated pulmonary inflammation. The decreased influx of neutrophils in response to IFN-β administration was accompanied by decreased expression of TNF-α, chemokines, and adhesion molecules in the lung. Although this is an interesting finding, one should keep in mind that IFN-β therapy may also worsen bacterial pneumonia [21], although until now only one case-report has been published. However, the increase in incidence of bacterial pneumonia was only described for a situation in which long-term treatment with IFN-β was applied [22]. IFN-β is a potent immunomodulator with diverse effects. Several studies have shown that IFN-β reduces the migration of inflammatory cells across the blood-brain barrier [12]. This is likely accomplished by reducing the expression of endothelial adhesion molecules, ICAM-1 and VCAM-1, and by downregulating the production of chemokines [23,24]. Although the immunomodulatory effects of IFN-β have been described in brain-related diseases including multiple sclerosis and focal cerebral ischemia, we are the first to describe the modulatory effects of IFN-β in pulmonary inflammation. The exact mechanisms by which IFN-β attenuates SAH-induced lung inflammation need to be further clarified. In our study, IFN-β was administered systemically; therefore it may be possible that IFN-β had a direct effect on pulmonary cells. For example, Kiss *et al*. showed that IFN-β treatment ameliorated vascular leakage in ALI via upregulation of 5'-nucleotidase (CD73) on pulmonary endothelial cells [25]. Although we did not look at CD73, it could be a possible explanation for our findings. IFN-β could also have an indirect effect by inhibiting either the activation of sympathetic nervous system or reducing the systemic pro-inflammatory environment and subsequently preventing the upregulation of pro-inflammatory mediators in the lung. Although we did not measure catecholamines, we could not see an effect of IFN-β treatment on TNF-α levels in the blood (data not shown). Finally, IFN-β could have a direct effect on cerebral inflammatory responses after SAH, thereby indirectly regulating the lung inflammation. Our preliminary data do not confirm this hypothesis since IFN-β did not have any effect on the SAH-induced cerebral inflammation (manuscript in preparation, Tiebosch *et al*.)

In conclusion, SAH induces a pro-inflammatory environment in the lung, which can be efficiently blocked by IFN-β. Therefore, our data strongly suggest that IFN-β may be an attractive clinical candidate to prevent SAH mediated lung inflammation.

## Conclusions

We show here that subarachnoid hemorrhage (SAH) results in the upregulation of pro-inflammatory mediators in the lung as well as recruitment of neutrophils into the lung. In addition, we report that treatment with IFN-β completely abolishes the SAH-induced pulmonary inflammation. Our data imply that SAH is associated with pulmonary inflammation and that IFN-β may be an attractive therapeutic candidate to prevent SAH-mediated lung inflammation.

## Key messages

• Subarachnoid hemorrhage in rats is associated with increased pulmonary inflammatory mediators.

• Subarachnoid hemorrhage results in increased neutrophil influx into the lung.

• IFN-β treatment completely abolishes the subarachnoid hemorrhage-induced pulmonary inflammation.

## Abbreviations

ALI: acute lung injury; CINC: cytokine-induced neutrophil chemoattractant; ECA: external carotid artery; ICA: interior carotid artery; ICAM: inter-cellular adhesion molecule; I/E: inspiration to expiration; IFN: interferon; MIP: macrophage inflammatory protein; MPO: myeloperoxidase; MV: mechanical ventilation; NPE: neurogenic pulmonary oedema; RT: reverse transcriptase; SAH: subarachnoid hemorrhage; VCAM: vascular cell adhesion molecule; VILI: ventilation-induced lung injury.

## Competing interests

The authors declare that they have no competing interests.

## Authors' contributions

PC performed the experimental work, interpreted the results and drafted the manuscript. IT and RZ performed the experimental work and were responsible for critical review of the manuscript. PM participated in study design and was responsible for critical review of the manuscript. RD, CH, JK and WB supervised the study, were involved in interpreting the results and correcting the manuscript. All authors have read and approved the final version of the manuscript.
